# COVID-19 and lipids. The role of lipid disorders and statin use in the prognosis of patients with SARS-CoV-2 infection

**DOI:** 10.1186/s12944-021-01563-0

**Published:** 2021-10-25

**Authors:** Stanisław Surma, Maciej Banach, Joanna Lewek

**Affiliations:** 1 Faculty of Medicial Sciences in Katowice, Medical University of Silesia in Katowice, Poland; Medyków 18, 40-752 Katowice, Poland; 2 Club of Young Hypertensiologists, Polish Society of Hypertension, Warsaw, Poland; 3 Department of Preventive Cardiology and Lipidology, Medical University of Lodz, Rzgowska 281/289, 93-338 Lodz, Poland; 4 Cardiovascular Research Centre, University of Zielona Gora, Zielona Gora, Poland; 5 Department of Cardiology and Adult Congenital Heart Diseases, Polish Mother’s Memorial Hospital Research Institute (PMMHRI), Rzgowska 281/289, 93-338 Lodz, Poland

**Keywords:** Statins, Dyslipidemia, SARS-CoV-2, COVID-19

## Abstract

The global coronavirus disease 2019 (COVID-19) pandemic caused by the SARS-CoV-2 coronavirus started in March 2020. The conclusions from numerous studies indicate that people with comorbidities, such as arterial hypertension, diabetes, obesity, underlying cardiovascular disease, are particularly vulnerable to the severe course of COVID-19. The available data also suggest that patients with dyslipidemia, the most common risk factor of cardiovascular diseases, are also at greater risk of severe course of COVID-19. On the other hand, it has been shown that COVID-19 infection has an influence on lipid profile leading to dyslipidemia, which might require appropriate treatment. Owing to antiviral, anti-inflammatory, immunomodulatory, and cardioprotective activity, statin therapy has been considered as valuable tool to improve COVID-19 outcomes. Numerous observational studies have shown potential beneficial effects of lipid-lowering treatment on the course of COVID-19 with significant improved prognosis and reduced mortality.

## Introduction

Severe acute respiratory syndrome coronavirus 2 (SARS-CoV-2) was first identified in December 2019 in Wuhan, China. As a coronavirus belonging to the family of zoonotic viruses, its genetic material is a single-stranded ribonucleic acid. SARS-Cov-2 causes the Coronavirus Disease 2019 (COVID-19), an acute infectious disease of the respiratory system, which was declared a global pandemic by the World Health Organization on March 11, 2020. COVID-19 is characterized by a mortality of about 2,3% and a transmission rate of about 2.5–3.0 (the number of newly infected people per previously infected person) [1]. Over 4.4 million people worldwide have died from COVID-19 so far (September 2021).

In the course of COVID-19, there are three phases which differ in the severity of the course, covering the time from early infection to recovery or death [2]. From a clinical point of view, the symptoms occurring after contracting COVID-19 are important. A meta-analysis of 15 studies by Lopez-Leon et al. assessed the incidence of long-term effects of COVID-19, wchich included: fatigue (58%), headache (44%), attention disorders (27%), hair loss (25%) and dyspnea (24%) [3]. Based on a meta-analysis of 38 studies conducted by Iqbal et al. it was found that fatigue and dyspnoea were the most prevalent symptoms in acute post-COVID-19 and fatigue and sleep disturbance in chronic post-COVID-19 syndrome [4].

A recently published meta-analysis of 27 studies by Shi et al. summarized our understanding of the factors that predict COVID-19 mortality. The most important predictors of death in the course of COVID-19 include: renal replacement therapy (relative risk: RR = 53.5; 95%CI: 22.4–127.3; *p* < 0.001), invasive mechanical ventilation (RR = 29.3; 95% CI: 21.5–39.9; *p* < 0.001), high level of procalcitonin (RR = 19.9; 95% CI: 7.31–53.9; *p* < 0.001), chronic kidney disease (RR = 8.37; 95%CI: 3.94–17.8; *p* < 0.001) and cerebrovascular diseases (RR = 7.66; 95% CI: 3.87–15.2; *p* < 0.001) [5]. The impact of cardiovascular diseases (CVD) on the prognosis of COVID-19 patients was assessed in a meta-analysis of 51 studies by Bae et al. It was shown that factors deteriorating the prognosis of COVID-19 (severe course or death) included: arterial hypertension (odds ratio: OR = 2.50; 95% CI: 2.15–2.90), diabetes (OR = 2.25; 95% CI: 1.89–2.69) and other CVD (OR = 3.11; 95% CI: 2.55–3.79) [6]. The results of the above studies indicate that CVD significantly worsens the prognosis of patients with COVID-19 [7]..

Taking into account the high prevalence of lipid disorders and their important role in shaping cardiovascular risk [8], a literature review was carried out on lipidological problems and COVID-19. Based on a review of the latest literature, the article describes a potential relationship between the incidence of lipid disorders and the prognosis of COVID-19 patients, the impact of SARS-CoV-2 infection on lipid metabolism and the role of statins in the treatment of COVID-19 patients. Some available literature data has been critically discussed and clinical recommendations regarding statin use during the COVID-19 pandemic have been provided. This article briefly and comprehensively presents the latest findings on lipidology and COVID-19. Moreover, the article also summarizes the latest guidelines for the use of statins in COVID-19.

### Influence of dyslipidemia on the course and prognosis in COVID-19 patients

Cholesterol plays an important role in SARS-CoV-2 entry into host cells [9]. In an in vitro study, the depletion of membrane-bound cholesterol in ACE2 (angiotensin converting enzyme-2)-expressing cells led to a reduced infectivity of SARS-CoV, since the binding of the spike protein was reduced by 50% [10]. In patients with dyslipidemia, the systemic cholesterol content is increased, which may lead to an increase in the number of ACE2 receptors in the lipid rafts of cells and facilitate the penetration of SARS-CoV-2 into them. The role of lipoproteins and their receptors in the penetration of SARS-CoV-2 into cells requires further biochemical research [9]. It has been suggested that lipids, including fatty acids, interact with SARS-CoV-2 and may be a potential intervention strategy against COVID-19 [11]. Moreover, it is indicated that cholesterol, by influencing SARS-CoV-2’s S spike configuration, may increase the affinity for ACE2 and thus the infectivity of this coronavirus [12]. An important role for the scavenger receptor, class B type 1 (SR-B1) in penetration of SARS-CoV-2 into the host cell has also been suggested. In experimental studies, it was found that the use of the SR-B1 antagonist reduced the infectivity of SARS-CoV-2 [13].

The relationship between lipid disorders, their treatment and the severity of COVID-19 has been an object of interest in some recently published observational studies, which indicate that dyslipidemia is associated with a poorer prognosis in COVID-19 patients. In a meta-analysis of 9 observational studies conducted by Atmosudigdo et al. which included 3663 patients with COVID-19, dyslipidemia occurred in 18% of patients. It was shown that dyslipidemia was associated with a 39% increase in the risk of mortality in COVID-19 patients (RR = 1.39; 95% CI: 1.02–1.88; *p* = 0.010). Moreover, dyslipidemia also increased the risk of severe course of COVID-19 by 39% (RR = 1.39; 95% CI: 1.03–1.87; *p* = 0.008). In the meta-regression analysis, it was found that such an adverse effect of dyslipidemia on the course and prognosis of COVID-19 was more pronounced in older men with concomitant arterial hypertension. The authors of this meta-analysis indicate that dyslipidemia may potentially be a factor that worsens the course and prognosis of COVID-19. However, there were lacking data concerning comorbidities and medications taken by patients [14]. Another meta-analysis of 7 studies by Hariyanto and Kurniawan including 6922 patients with COVID-19 showed similar association between dyslipidemia and the course of COVID-19. The authors proved that dyslipidemia can possibly increase the risk of severe COVID-19 by 39% (RR = 1.39; 95% CI: 1.03–1.87; *p* = 0.03) [15]. Likewise, Santos et al. in a study involving 3711 patients with COVID-19, showed that dyslipidemia was associated with an increased risk of a severe course of COVID-19 (OR = 12; 95% CI: 1.33–108; *p* = 0.03) [16]. This was also supported by Choi et al. in the meta-analysis which showed that patients with COVID-19 and dyslipidemia were characterized by 49% (RR = 1.49; 95% CI: 1.11–2.01; *p* = 0.01) higher risk of severe COVID-19 than those with normal lipid profile. On the other hand, it was shown that the increased concentration of total cholesterol, low density lipoprotein (LDL), high density lipoprotein (HDL) and triglycerides in the serum was inversely correlated with the severity of COVID-19 [17]. A recently published meta-analysis of 28 studies by Liu et al. summarized the existing data on the impact of dyslipidemia on the prognosis of patients with COVID-19. The study included 12,995 patients with COVID-19. Dyslipidemia has been shown to increase the risk of severe course of COVID-19 (OR = 1.27, 95%CI: 1.11–1.44, *p* = 0.038) and the risk of death due to COVID-19 (OR = 2.13, 95%CI: 1.84–2.47, *p* = 0.001). The researchers conclude that the occurrence of dyslipidemia in patients with COVID-19 may worsen their prognosis [18].

A study by Masana et al.*,* involving 1411 patients with COVID-19, assessed the usefulness of serum total cholesterol, LDL, non-HDL, HDL cholesterol and triglicerydes to predict the COVID-19 prognosis (severe versus mild). It has been shown that low HDL and high triglycerides level measured before or during hospitalization were strong predictors of severe COVID-19. Researchers indicate that the lipid profile should be considered a sensitive marker of inflammation and should be measured in COVID-19 patients [19]. An interesting study by Yıldırım and Kaya found that a plasma atherogenic index (AIP) greater than 0.6285 was a predictor of in-hospital mortality in patients with COVID-19 and an early biomarker of severe disease [20].

The opposite results were obtained by Petrilli et al. who assessed the impact of dyslipidemia on the risk of hospitalization and the severe course of COVID-19 in 5279 patients. It has been shown that the occurrence of dyslipidemia was not associated with an increased risk of hospitalization (OR = 0.93; 95% CI: 0.75–1.2; *p* = 0.51) or an increased risk of mortality in COVID-19 (OR = 0.98; 95% CI: 0.82–1.17; *p* = 0.79) [21]. It was then confirmed by Chang et al. in a retrospective study of 211 patients with mild COVID-19 symptoms. They found that dyslipidemia was not associated with an increased risk of progression to severe COVID-19 (OR = 1.203; 95% CI: 0.010–148.987; *p* = 0.940) [22]. Similarly, Simonnet et al. found no relationship between dyslipidemia and the course of COVID-19 (OR = 0.68; 95% CI: 0.24–1.97; *p* = 0.48) [23]. The impact of overweight/obesity as well as dyslipidemia on the risk of starting artificial ventilation was assessed in 124 patients. There was a significant relationship between weight gain (assessed using body mass index; BMI) and the risk of worsening the course of COVID-19, but no such relationship was found for dyslipidemia (OR = 0.68; 95% CI: 0.24–1.97; *p* = 0.48) [23]. The discrepancies between the results of the above studies may be due to several reasons. First, the criteria for dyslipidemia diagnosis were different between studies (a huge difference in the incidence of dyslipidemia between studies conducted in the United States and China was observed) [17]. Secondly, it was found that in the course of SARS-CoV-2 infection, there are rapid changes in lipid metabolism, which makes it difficult to interprete whether dyslipidemia occurred before or during COVID-19 [17, 24]. Thirdly, based on the results of the above studies, it can be assumed that perhaps dyslipidemia in its own does not increase the risk of the severe course of COVID-19, but serves as a component of diseases that increase such risk, such as obesity or type 2 diabetes, thus may affect the overall prognosis. Especially, diabetes significantly increases the risk of severe course and death from COVID-19. However, a systematic review by Choi et al. showed that probably not dyslipidemia itself, but CVD caused by it, worsen the prognosis in COVID-19 [17]. To sum up, the relationship between the prevalence of dyslipidemia and COVID-19 requires further studies.

A meta-analysis of 13 studies by Moazzami et al. assessed metabolic risk factors for severe COVID-19. It has been shown that the incidence of obesity and diabetes in patients with COVID-19 was 29% (95% CI: 14–47%) and 22% (95% CI: 12–33%), respectively [25]. A study by Caussy et al. showed that the incidence of obesity depended on the course of COVID-19. Using the standardized prevalence index, the authors found that obesity (BMI ≥ 30 kg / m^2^) was more common in patients with severe and critical COVID-19 compared to the general population (standardized prevalence ratio: PR = 1.35; 95% CI: 1.08–1.66 and PR = 1.89; 95% CI: 1.33–2.53) [26]. A meta-analysis of 20 studies by Faghir-Gangi et al. which included 5515 patients with COVID-19, showed that type 2 diabetes was present in 14% (95% CI: 11–17%) [27]. The presence of both obesity and type 2 diabetes worsened the prognosis of COVID-19 patients. In a meta-analysis of 75 studies by Popkin et al. the impact of obesity on the prognosis of COVID-19 patients was assessed. Obesity has been shown to increase the risk of hospitalization for COVID-19 by 113% (OR = 2.13; 95% CI: 1.74–2.60; *p* < 0.0001), the risk of being admitted to the intensive care unit by 74% (OR = 1.74; 95% CI: 1.46–2.08; *p* < 0.0001), a 66% risk of the need for artificial ventilation (OR = 1.66; 95% CI: 1.38–1.99; *p* < 0.0001) and a 48% risk of death from COVID-19 (OR = 1.48; 95% CI: 1.22–1.80; *p* < 0.001) [28]. The relationship between diabetes and COVID-19 severity and mortality was assessed in a meta-analysis of 33 studies by Kumar et al. It was shown that diabetes significantly increased the risk of severe COVID-19 (OR = 2.75; 95% CI: 2.09–3.62; *p* < 0.01) and increased the risk of death from COVID-19 (OR = 1.90; 95% CI: 1.37–2.64; *p* < 0.01) [29]. Dyslipidemia frequently occurs in obese or diabetic patients. In a study by Kaur and Aeri involving 150 obese patients, it was shown that dyslipidemia occurred in 78% patients [30]. On the other hand, a study by Li et al. which involved 9285 patients with type 2 diabetes showed that dyslipidemia occurred in 59.3% of them [31].

In general, at the moment it is not known whether dyslipidemia increases the risk of severe course and death from COVID-19. However, dyslipidemia frequently occurs in obese and/or type 2 diabetic patients and requires optimal therapy. On the other hand, both obesity and type 2 diabetes are common in COVID-19 patients and significantly worsen their prognosis. As a result, an exact relationship between the incidence of dyslipidemia and the prognosis of COVID-19 patients requires further studies.

Regardless of the above inconsistent results of observational studies on the influence of dyslipidemia on the course of COVID-19, it has been shown that lipid metabolism is disturbed in the course of this disease [24]. Moreover, it was found that the use of statins improved the prognosis of patients with COVID-19 [32].

### Influence of COVID-19 on lipid metabolism

**Fig. 1:**
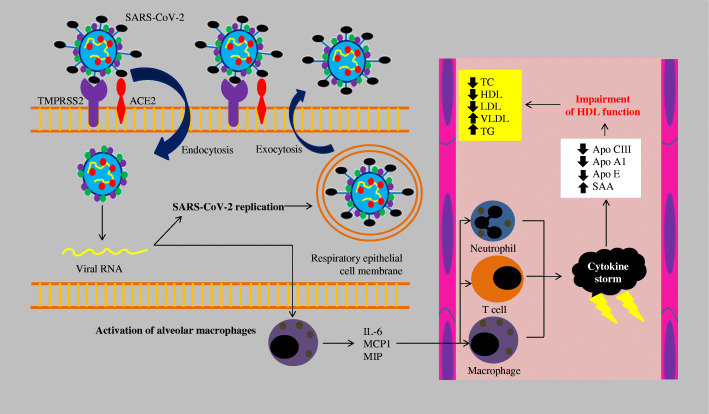
Lipid disorders induced by SARS-CoV-2 infection [33]. SARS-CoV-2 - severe acute respiratory syndrome coronavirus 2; TMPRSS2 - transmembrane serine protease 2; ACE2 - angiotensin-converting enzyme 2; RNA - ribonucleic acid; IL-6 – interleukin 6; MCP1 - monocyte chemoattractant protein-1; MIP - macrophage inflammatory proteins; Apo CIII - apolipoprotein C-III; Apo A1 – apolipoprotein A1; Apo E – apolipoprotein E; SAA - serum amyloid A; TC – total cholesterol; HDL – high-density lipoprotein; LDL – low-density lipoprotein; VLDL – very-low-density lipoprotein; TG – trigliceryde

In the course of COVID-19, a temporary disturbance of lipid metabolism was observed, mainly caused by impairment of HDL function (Fig. 1) [33]. The SARS-CoV-2 coronavirus binds to ACE2 via the spike protein (S), which allows entry into the cell. The penetration of SARS-CoV-2 into the lung tissue leads to the activation of alveolar macrophages, which in turn leads to the release of inflammatory mediators, such as: interleukin 6 (IL6), monocyte chemoattractant protein-1 (MCP1) and macrophage inflammatory protein (MIP). These proteins attract further macrophages, neutrophils, and T lymphocytes. The activation of immune system cells leads to the development of uncontrolled inflammation, the so-called cytokine storm and dysregulation of the immune system, as well as the accumulation of eicosanoids, such as: prostaglandin E2 (PGE2), thromboxane B2 (TXB2), leukotriene B4 (LTB4) and lipoxin A4 (LXA4). The uncontrolled inflammation leads to the impairment of HDL lipoprotein function by reducing the concentration of apolipoprotein AI (ApoA-I), apolipoprotein E (ApoE) and increasing the concentration of serum amyloid A (SAA). These changes reduce the anti-inflammatory, antioxidant, and immunomodulatory properties of HDL lipoproteins. Oxidized HDL and LDL lipoproteins (oxLDL and oxHDL) are potent activators of the oxidized LDL scavenge receptor (LOX-1), causing further inflammation and tissue damage. The extracellular portion of serum-soluble LOX-1 (sLOX-1) further stimulates the interaction between oxidized lipids and circulating macrophages, releasing pro-inflammatory cytokines such as IL-6, interleukin 10 (IL-10) and tumor necrosis factor alpha (TNF-α). Impaired function of the enzyme paraoxonase 1 (PON1) located on the surface of HDL lipoprotein and an excessive inflammatory response leads to further lipid oxidation. Increased percentage of oxidized oxLDL and oxHDL lipoproteins leads to the impairment of cholesterol re-transport characterized by insufficient interaction of ApoA-I with the ATP-binding cassette transporter (ABCA1) on macrophages and decreased esterification of cholesterol by lecithin cholesterol acyltransferase (LCAT). The pathophysiological effect of this is reduced return of cholesterol esters to the liver immediately after interaction with hepatic SR-B1 or indirectly after transfer to LDL by cholesterol ester transfer protein (CETP) and uptake by hepatic LDL receptors (LDL-R). Low concentrations of ApoE and apolipoprotein C-III (ApoC-III) on HDL reduce the activity of lipoprotein lipase (LPL), which in turn leads to the accumulation of very low-density lipoproteins (VLDL) and triglycerides. It is also worth mentioning that oxidized phospholipids in LDL lipoproteins are recognized as danger-associated molecular patterns (DAMPs), which leads to inflamosome stimulation and impaired vascular endothelial cell function and atherosclerosis progression. The effects of the interaction of oxLDL and LOX-1 (accumulation of oxLDL inside the cells) also contribute to the accelerated atherosclerosis progression [33].

The characteristics of lipid metabolism disorders in patients with COVID-19 has been partially confirmed in observational studies (Tab. 1). However, caution should be exercised regarding the results of observations indicating a hypolipidemia initiation in patients with COVID-19 and its impact on the prognosis of this disease. In the commentary to the study by Wei et al. [35] a number of limitations of this type of observation were indicated. It was found that a single assessment of the lipid profile (on admission to the hospital) did not predict the influence of lipid concentration fluctuations on the course of COVID-19. The authors of the comment also state that the adopted criterion of hypolipidemia is not correct. In addition, the effect of lipid-lowering therapy on the results in this study was not included. Thus, the authors of the comment conclude that more analyzes, and studies are needed to establish the relationship between low LDL cholesterol and the course of COVID-19 [39].

**Table 1:** Effect of SARS-CoV-2 infection on serum lipid concentrations

Author/ year	Number of subjects	Total cholesterol [mg/dl]		LDL [mg/dl]		HDL [mg/dl]		TG [mg/dl]		Conclusions
		COVID-19 (+)	COVID-19 (−)	COVID-19 (+)	COVID-19 (−)	COVID-19 (+)	COVID-19 (−)	COVID-19 (+)	COVID-19 (−)	
Hu et al., [34]; 2020	114 - COVID-19 (+)80 – COVID-19 (−)	152*	188	85.4*	119	42.1*	49.5	102	187	HDL was significantly lower in severely ill group compared to moderate group. Total cholesterol, LDL, and TG were not different
Wei et al., [35]; 2020	597 - COVID-19 (+)40 - COVID-19 (−)	169*	184	88*	110	49**	52	No data	No data	HDL was decreased significantly in critically ill but not in severely ill patients. LDL was significantly decreased in both severely and critically ill patients
Wang et al., [36]; 2020	229 - COVID-19 (+)1140 - COVID-19 (−)	147**	181	103*	110	30.4**	53.4	95*	106	HDL were lower in severely ill patients. Total cholesterol, LDL, and triglycerides were not significantly different in severely ill patients
Wang et al., [37]; 2020	143 - COVID-19 (+)	No data	No data	101	No data	35.1	No data	No data	No data	HDL were lower in severely ill patients. LDL were not significantly different in severely ill patients
Zhang et al., [38]; 2020	74 - COVID-19 (+)***	164	No data	101	No data	40.2	No data	111	No data	HDL and LDL were decreased in severely ill patients

* *p* < 0.001; * * *p* < 0.05; *** patients with type 2 diabetes

**Fig. 2:**
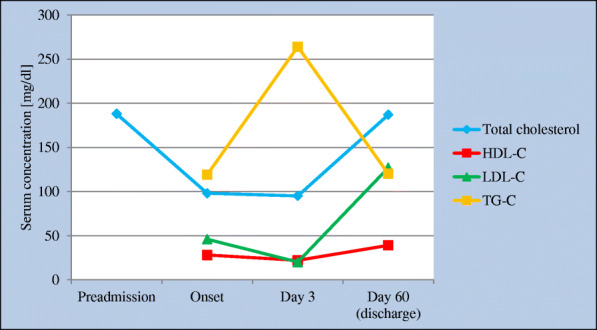
Lipid profile during COVID-19 – adapted from a paper by Sorokin et al. [33]

Moreover, in the study by Sorokin et al. describing a clinical case of a patient with COVID-19, it was shown that the concentrations of individual serum lipids changed temporarily depending on the disease duration (Fig. 2) [33]. Similar results regarding the level of LDL were obtained by Fan et al. in a study involving 17 patients with COVID-19 [40]. In a study by Li et al. the relationship between changes in serum lipid levels and the prognosis of patients with COVID-19 was assessed. The study included 424 patients with a severe course of COVID-19 (34 survivors and 390 non-survivors). It was shown that during hospitalization LDL, total cholesterol, HDL and ApoA1 showed an increasing trend in survivors but showed a downward trend in non-survivors. Moreover, the serum concentrations of HDL and ApoA1 were inversely correlated with C-reactive protein (CRP), length of hospital stay of survivors, and disease severity scores. Patients with high ratios of CRP/ HDL (> 77.39) or CRP / ApoA1 (> 72.37) had statistically significantly higher mortality rates during hospitalization. The researchers conclude that during severe COVID-19, HDL and ApoA1 concentrations are dramatically decreased in non-survivors. Moreover, high CRP/HDL ratio is significantly associated with an increased mortality and poor prognosis [24]. In the study by Caterino et al. the lipidomic profile and the profile of proinflammatory cytokines and alarmins in COVID-19 patients were analyzed. A significant role of adipose tissue has been demonstrated (in the pathogenesis of lipid disorders observed in patients with COVID-19 [41]. It is worth mentioning that there are ideas addressing the potential role of COVID-19 in the pathogenesis of diabetes. The risk of permanent damage to pancreatic β-cells by SARS-CoV-2 has been suggested. However, this concept requires further research [42].

Thus, during COVID-19 there are numerous changes in the lipid profile which correlate with the severity of the course of this disease. It should be emphasized that the observed relationship between the severity of COVID-19 and the decrease in lipid levels may not be the cause of poor prognosis, but a consequence of acute inflammatory disease. The pathophysiology of lipid metabolism disorders in patients with COVID-19 has not been fully understood so far. It seems that an important factor disturbing HDL function is the increase serum SAA concentration under the influence of cytokine storm. It has been described that inflammation alters hepatic apolipoprotein gene expression and promotes binding of the pro - inflammatory SAA which, in turn, displaces and decreases ApoA1 concentrations in HDL [43]. Moreover, it was shown that the concentration of SAA in the serum positively correlated with the severity of the course of COVID-19 (*p* < 0.01) [44].

It is worth mentioning that the lipid disorders observed in patients with COVID-19 are similar to those observed in the course of other infections and inflammatory diseases [45]. In summary, in the course of COVID-19, lipid disorders (very often a decrease in total cholesterol, HDL and LDL, and an increase in triglycerides) caused by inflammation may occur.

### Statins and COVID-19: the results form the clinical studies

The effect of statin use on the severity and prognosis of COVID-19 has been the subject of several meta-analyses (Tab. 2.). A study by *Lee* et al. involving 10,448 COVID-19 patients investigated the effect of statin administration on COVID-19 mortality. Moreover, the effect of statins on the risk of death was compared with a retrospective cohort of patients with pneumonia. Researchers showed a significant decrease in hazard ratio (HR) associated with the use of statins (HR = 0.637; 95% CI: 0.425–0.953; *p* = 0.0283). Moreover, when comparing the HR between COVID-19 patients and the retrospective cohort of hospitalized pneumonia patients, the use of statins showed similar benefits. Thus, the use of statins correlated significantly with lower mortality in patients with COVID-19, consistent with the findings in patients with pneumonia [59].

**Table 2:** Summary of the results of meta-analysis of studies assessing the effect of statins on the course and prognosis of COVID-19. ICU – intensive care unit

Author/ year	Analyzed period	Number of included studies	Sample size	Results	Conclusions
Kow and Hasan [46]	to July 27, 2020	4	8990	Severity and mortality: risk ↓30% (HR = 0.70; 95% CI: 0.53–0.94)	The use of statins improves the prognosis in patients with COVID-19
Scheen [47]	Dec 2019 to Dec 2020	13	42,722	Mortality: no statistically riskreduction (OR = 0.97; 95% CI: 0.92–1.03)Severity: no statistically risk reduction (OR = 1.09; 95% CI: 0.99–1.22) Adjusted for confounders, a 27% reduction in the risk of severe disease and mortality in COVID-19 was demonstrated (adjusted OR = 0.73 ± 0.31 versus unadjusted OR = 1.44 ± 0.84; *p* = 0.0028).	Statin therapy in patients with COVID-19 may improve their prognosis
Onorato et al. [48]	2019 toSep 28, 2020	7	2398	Severity and mortality: risk ↓ by 41 (OR = 0.59; 95% CI: 0.35–0.99). The analysis of the results of studies in which statins were used before admission to the hospital showed even greater benefits of their use (OR = 0.51; 95% CI: 0.41–0.64).	The use of statins improves the prognosis in patients with COVID-19
Pal et al. [49]	to Dec 18, 2020	14	19,988	Severity and mortality: no statistically risk reduction (OR = 1.02; 95% CI: 0.69–1.50). After adjustment reduce the risk of adverse outcomes by 49% (OR = 0.51; 95% CI: 0.41–0.63).	Statin therapy in patients with COVID-19 may improve their prognosis
Chow et al. [50]	Jan 2019 to Dec 2020	13	110,078	The use of statins before hospitalization: no significantly affect the risk of death (OR = 0.62; 95% CI: 0.38–1.03). The use of statins since the diagnosis of COVID-19: reduced the risk of death (OR = 0.57; 95% CI: 0.43–0.75). The use of statins did not reduce the mortality of COVID-19 patients admitted to the ICU (OR = 0.65; 95% CI: 0.26–1.64). Among patients in non-ICU, statin users were at lower risk of death (OR = 0.64; 95% CI: 0.46–0.88). The use of statins did not reduce the risk of admission to an ICU	Patients administered statins after COVID-19 diagnosis or non-ICU admitted patients were at lower risk of mortality
Vahedian-Azimi et al. [32]	to Nov 2, 2020	24	32,715	Significant reductions risk of ICU admission (OR = 0.78; 95% CI: 0.58–1.06). No significant effect on risk of tracheal intubation (OR = 0.79; 95% CI: 0.57–1.11). Significant reductions of death (OR = 0.70; 95% CI: 0.55–0.88). Was demonstrated that decrease mortality by in-hospital application of statins (OR = 0.40; 95% CI: 0.22–0.73), compared with pre-hospital use (OR = 0.77; 95% CI: 0.60–0.98)	Statins potentially reduction of ICU admission and total mortality reduction in COVID-19 patients
Wu et al [51].	to Nov 10, 2020	28	63,537	Statin use was associated with a reduction in the risk of mortality (OR = 0.71, 95% CI: 0.55–0.92) and the need for artificial ventilation (OR = 0.81, 95% CI: 0.69–0.95). Statin use was not found to reduce the risk of treatment in ICU (OR = 0.91; 95% CI: 0.55–1.51).	Statins can improve the prognosis of COVID-19 patients, so it does not seem necessary to stop taking them when the patient is admitted to the hospital.
Permana et al [52].	Dec 1, 2019 to Nov 11, 2020	13	52,122	In-hospital use of statin was associated with a decreased risk of mortality by 56% (RR = 0.54; 95% CI: 0.50–0.58).Pre-admission use of statin was not associated with risk of mortality (RR = 1.18; 95% CI: 0.79–1.77).	In-hospital use of statins was associated with a decreased risk of death in patients with COVID-19
Yetmar et al. [53]	to Dec, 2020	16	395,513	The use of statins before contracting COVID-19 reduced the risk of mortality (adjusted RR = 0.65; 95% CI: 0.56–0.77) and the risk of severe disease (aRR = 0.73; 95% CI: 0.57–0.94).	The use of statins is associated with a lower risk of death or serious illness in patients with COVID-19. The important role of the continued use of statins in patients indicated for lipid-lowering therapy during the COVID-19 pandemic is indicated.
Hariyanto and Kurniawan [54]	to Nov 25, 2020	35	11,930,583	Statin use has not significant effect on reducing the risk of COVID-19 (OR = 1.09; 95% CI: 0.58–2.03). Statin use has not significant effect on reducing the risk severity course of COVID-19 (OR = 1.07; 95% CI: 0.86–1.33).	Statin use did not improve the outcomes of patients with COVID-19.
Zein et al. [55]	to March 1, 2021	8	14,446	The use of statins reduced the risk of mortality (RR = 0.72; 95% CI: 0.55–0.95). In the subgroup of patients who used statins during hospitalization, an even lower risk of mortality was observed (RR = 0.71; 95% CI: 0.54–0.94). The observed effects were not influenced by such factors as: age, male gender, diabetes and arterial hypertension.	Statins reduce the risk of mortality in COVID-19 patients.
Diaz-Arocutipa et al. [56]	to March 3, 2021	25	147,824	In-hospital use of statins did not affect the risk of mortality (adjusted HR = 0.74; 95% CI: 0.49–1.10). Chronic statin use significantly reduced the risk of mortality (aHR = 0.71; 95% CI: 0.56–0.91).	Statins, especially when used chronically, reduce the risk of death in patients with COVID-19
Kollias et al. [57]	to March 5, 2021	22	114,688	Using statins versus not using statins reduced the risk of mortality (HR = 0.65; 95% CI: 0.53–0.81 and OR = 0.65; 95% CI: 0.55–0.78).	Statin treatment was associated with an approximately 35% reduction in adjusted risk of death in hospitalized COVID-19 patients.
Kow and Hasan [58]	to Jun 3, 2021	35	138,402	Statin use reduced the risk of mortality from any cause (OR = 0.63, 95% CI: 0.51–0.79), and the risk of severe COVID-19 (OR = 0.80, 95% CI: 0.73–0.88).	The use of statins is associated with a better prognosis in COVID-19 patients.

Overall, the results of the metaanalysis remain inconsistent when assessing the effect of statins on the improvement of prognosis in COVID-19 patients. Perhaps the conflicting results are an effect of confounding factors such as age, gender, comorbidities, polypharmacy, genetic predisposition, environmental factors, lifestyle, etc. An important factor that could explain divergent metaanalysis results could be the difference in the type of statin used. This possibility was highlighted in a study by Rossi et al. which showed that administration of simvastatin and atorvastatin reduced mortality in COVID-19 patients, whereas those treated with pravastatin and rosuvastatin did not show such a difference [60]. A study by Cariou et al. indicates that the effect of statins may depend on the cardiovascular burden (stage, severity of the underlying disease and comorbidity) of COVID-19 patients [61]. Interpretation of the results of the presented metaanalysis should be exercised with caution, as this type of research is burdened with errors [62]. Moreover, there is a discussion on the methodology used in some meta-analyzes [63, 64]. Future studies should provide more information on the possible benefits of statin treatment in COVID-19 patients. It is known, however, that statin therapy should not be discontinued in patients with COVID-19 [65].

The causal relationship between statin use and the prognosis of COVID-19 patients can only be confirmed by the results of randomized controlled clinical trials (RCTs). A comprehensive review of the literature by Talasaz et al. summarized the ongoing RCTs on statin use (especially atorvastatin and rosuvastatin) in treatment of COVID-19. Moreover, the authors indicate that the role of OMEGA-3 fatty acid, fibrates, and niacin in the treatment of COVID-19 is also under investigation [66]. It should be emphasized that statins will not replace other drugs used in the treatment of COVID-19 patients. Statins can complement therapy in some patients.

It appears that the use of statins in patients with COVID-19 may also contribute to the reduction of the risk of lipid disorders observed in the long-term follow-up of patients infected with other SARS coronaviruses [67].

### Statins and COVID-19: mechanism

The beneficial effects of statin administration in COVID-19 patients have been observed in some studies due to their pleiotropic mechanism of action. Several mechanisms by which statins have a positive effect on the prognosis of COVID-19 patients have been described [68]. There are direct and indirect mechanisms of statin action in relation to SARS-CoV-2 infection [69].

#### Direct effect of statins

Cell membrane cholesterol is involved in the penetration of SARS-CoV-2 into the cell. As shown in Fig. 1, the process of SARS-CoV-2 penetration into the host cell requires the presence of the ACE2 protein. The presence of lipid rafts, which are subdomains of the cell membrane and containing significant amounts of cholesterol, has been shown to play an important role in promoting viral infections [7]. Lipid rafts are important in the interaction between the SARS-CoV-2 spike protein (S) and ACE2 and in virus endocytosis into cell [7]. The important role of ACE2 in coronavirus infections has already been demonstrated in the case of SARS-CoV-1. It was shown that ACE2-knockout mice infected with SARS-CoV-1 had significantly lower viral replication, S protein RNA levels and lung damage compared to wild-type mice with normal ACE2 expression [70]. Another confirmation of the significant role of lipid rafts in SARS-CoV-2 infection are the results of the study by Lu et al. The authors showed that ACE2 was co-localized in lipid rafts biomarkers, i.e., caveolin-1 and monosialotetrahexosyl ganglioside (GM1). Moreover, ACE2 was found to have been transferred to an environment other than the raft after reducing the cholesterol content of the lipid raft [71]. Interestingly, the penetration of SARS-CoV-2 into the cell may be mediated by proteins such as caveolins, clathrins, and dynamin located in lipid rafts [72]. The significant role of cholesterol in coronavirus infection was additionally supported by the study of the effect of cholesterol depletion in SARS-CoV infection, which resulted in a significant reduction of viral mRNA inside the cell [71]. By reducing endogenous cholesterol synthesis, statins reduce its amount in lipid rafts, which may limit the penetration of SARS-CoV-2 into the host cell [7]. Another direct important mechanism of action of statins is direct inhibition of SARS-CoV-2 replication. In silico studies it was shown that pitavastatin, rosuvastatin, lovastatin, and fluvastatin showed high affinity to the main protease SARS-CoV-2 (Mpro), which is involved in the regulation of replication and transcription of this virus [73]. The most important polymerase responsible for the RNA replication of the SARS-CoV-2 coronavirus is RNA-dependent RNA polymerase (RdRp). In a study by Baby et al. it was shown that pitavastatin strongly binds to the active site of this enzyme, as demonstrated by the simulation of molecular dynamics. The authors indicate that the demonstrated mechanism may be used in the treatment of SARS-CoV-2 infection [74].

Thus, statins may exert a direct inhibitory effect on SARS-CoV-2 penetration into the cell and its multiplication. These mechanisms require confirmation in vitro.

#### Indirect effect of statins

Statins reduce the overexpression of pro-inflammatory cytokines (may reduce the severity of the cytokine storm accompanying COVID-19). The most important cytokine involved in the cytokine storm in COVID-19 is IL-6. It has been shown that the level of IL-6 positively correlates with the severity of COVID-19 [75]. High levels of IL-6 in serum may contribute to the development of a cytokine storm, in addition to the cytokine storm, and the macrophage activation syndrome (MAS), a severe inflammation caused by activated macrophages, manifested by fever, hyperferritinemia, hypofibrinogenemia, coagulopathy and cytopenia [76]. Previous studies have shown that statins reduce IL-6 levels. A meta-analysis of 19 randomized clinical trials by Bonsu et al. including 6214 patients with heart failure, showed that statins are able to lower serum levels of both IL-6 and CRP, with a clear predominance of lipophilic statins, eg. atorvastatin, simvastatin and pitavastatin [77]. The mechanism of action of statins responsible for lowering the level of IL-6 is complex and consists of inhibiting the toll-like receptor 4 (TLR-4) and thus the pro-inflammatory action of the nuclear factor kappa B (NFκB) [69]. In mouse cells, atorvastatin was shown to reduce TLR-4 gene expression [78]. The effect of statins in reducing the risk of MAS is so far poorly understood and requires further research [69].

It is known that the vascular endothelium is damaged during COVID-19, therefore an interesting effect of statins is their effect on the vascular endothelium. It has been shown that statins protect the vascular endothelium from free radicals [69], reduce the pro-inflammatory activity of the NOD-like receptor family, pyrin domain containing 3 inflammosome (NLRP3) [79] and maximize the regenerative capacity of the vascular endothelium by increasing the level of human endothelial progenitor cells (EPCs) [80].

Another thing worth mentioning are the anticoagulant properties of statins. Thromboembolic complications are relatively common in COVID-19 patients. A multicentre retrospective study found the overall thrombotic complication rate associated with COVID-19 is 9.5% (95% CI: 6.8–12.8) [81]. Previous studies have shown that the use of statins (especially atorvastatin and rosuvastatin) reduced the risk of recurrent pulmonary embolism, which is one of the most serious thromboembolic diseases [82]. The explanation for the above beneficial effects of statins is their influence on the level of plasminogen activator inhibitor-1 (PAI-1). A meta-analysis of 16 randomized controlled trials conducted by Sahebkar et al. showed that statins (especially atorvastatin) significantly decreased the level of plasminogen activator inhibitor-1 (PAI-1) in the serum, thus increasing the degradation of fibrin clots by the enzyme plasmin [83]. It has also been shown that statins have an anticoagulant effect by reducing the plasma level of von Willebrand factor antigen [84].

The anti-fibrotic effect of statins seems to be very interesting from the point of view of complications of SARS-CoV-2 infection (especially in the long-COVID-19 syndrome). In a study by Li et al. which included 107 patients with COVID-19, it was shown that after 3–6 months after recovery, some of them may develop pulmonary fibrosis [85]. In an experimental study using mice and human lung fibroblasts/myofibroblasts, the effect of atorvastatin on the processes of fibrosis was assessed. In mice, administration of atorvastatin has been shown to reduce the number of fibrosis and collagen accumulation in an interstitial tissue, as well as reduced the levels of alpha-smooth muscle actin (α-SMA), lysyl oxidase-like protein 2 (LOXL2) and p-Src. In vitro studies have shown a reduction in α-SMA and fibronectin levels by limiting the action of transforming growth factor beta (TGF-β) [86]. It has also been suggested that statins inhibit the epithelial-mesenchymal transition (EMT) by attenuating TGF-β signaling known to be associated with post-infectious pulmonary fibrosis, causing remodeling and deposition of connective tissue among fibroblasts and epithelial cells [87, 88]. Statins also increase fibroblast apoptosis [89].

It is also worth mentioning that statins, by increasing the level of HDL lipoproteins, on average can have antiviral effects in this way. It has been shown that HDL lipoproteins can bind lipopolysaccharide as well as lipoteichoic acid [90, 91]. Moreover, HDL binding to lipopolysaccharide protects animals from the toxicity of this endotoxin [92]. Moreover, HDL can block the penetration of some viruses into cells, reducing their infection and multiplication in various tissues [93]. Moreover, HDL lipoproteins are characterized by antioxidant, anticoagulant, immunomodulating and anti-inflammatory properties, and also participate in the regeneration of the vascular endothelium [94]. The observed reduction in HDL lipoprotein levels by 40–70% in inflammatory diseases, including COVID-19, may further worsen the course of the disease [94].

A very interesting indirect antiviral mechanism of statin action is the effect of these drugs on arachidonic acid levels. A review of the literature by Hoxha concluded that arachidonic acid deficiency may increase the risk of developing COVID-19 [95]. A review of the literature by Das even points to the potential role of arachidonic acid in the prevention and treatment of COVID-19 [96]. A study by Risé et al. showed that statins significantly increase the concentration of arachidonic acid in the plasma in patients with hypercholesterolemia [97]. In an in vitro study by Goc et al., the effect of polyunsaturated ω-3 fatty acids (including arachidonic acid) on penetration of SARS-CoV-2 into the cell interior was assessed. These acids have been shown to interfere with the binding of SARS-CoV-2 to ACE2 on the cell surface [98]. Thus, statins may make it difficult for SARS-CoV-2 to infect cells by increasing arachidonic acid synthesis.

**Fig. 3:**
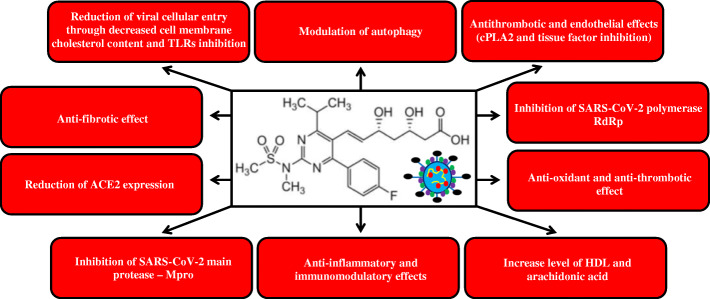
Summary of the direct and indirect mechanism of action of statins in the context of COVID-19. TLRs- toll like receptors; ACE2 - angiotensin converting enzyme 2; cPLA2 - phospholipase A2; TLR - toll-like receptor; HDL – high density lipoprotein

Thus, statins have many effects, both direct and indirect, that can improve the prognosis of COVID-19 patients. The mechanisms of beneficial effects of statin administration in COVID-19 patients are presented in Fig. 3.

### Clinical recommendations for patients with familial hypercholesterolemia

Considering that patients with familial hypercholesterolemia (heterozygous and homozygous) may be at increased risk of a severe course of COVID-19, Banach et al. developed brief recommendations for the treatment of this group of patients during the COVID-19 pandemic. The authors of the recommendation indicate that there is a number of scientific evidence indicating the potential beneficial effect of statins in reducing the severity of COVID-19. Patients with familial hypercholesterolemia should especially respect the therapeutic principles and social distancing, as their cardiovascular risk is much higher than in the general population. Treatment control should be done using telemedicine services, and medications should be prescribed for a longer period of time. In the event of a new diagnosis of familial hypercholesterolemia, the patient should be immediately referred to a specialist center for telephone advice and treatment. The use of statins in patients with COVID-19 is generally safe. All patients who require regular lipid apheresis should have access to this procedure [99]. emphasizing the need for intensive lipid-lowering therapy in that particular group of patients. As indicated by the guidelines of the European Society of Cardiology (ESC), the use of statins in the course of COVID-19 is safe in the majority of patients and may improve the prognosis of patients. Therefore, stopping statin therapy is not recommended [100]. It is woth emphasizing, that based on multiple data available on the role of statin therapy in COVID-19 patients in the just released Polish guidelines on the diagnosis and therapy of lipid disorders, for the first time in the world, the experts made an attempt at recommendations on the use of statins in patients with COVID-19 (Tab 3**.**).

**Table 3:** Polish recommendations for the treatment of lipid disorders in patients with COVID-19 [101]

Recommendations	Class	Level
In patients with COVID-19, the treatment of elevated LDL cholesterol levels should be optimized as soon as possible, especially in people with high or very high cardiovascular risk, who should use the highest recommended doses of statins.	**IIa**	**C**
The initiation or intensification of lipid lowering therapy and its monitoring is also possible during the e-visit/e-advice.	**I**	**C**
Optimal control of CVD risk factors, including in particular achieving therapeutic targets for LDL-C, during a pandemic is of special importance due to the need to reduce the risk of cardiovascular events and death in patients with COVID-19, in conditions of limited availability of health resources.	**I**	**C**
In people with COVID-19, optimal statin treatment should be continued, also during hospitalization, as it may be associated with an improved prognosis.	**IIa**	**B**

## Discussion

The issues of the impact of lipid disorders on the prognosis in COVID-19, the impact of COVID-19 on lipid metabolism and the role of statins in improving the prognosis of COVID-19 patients presented in this article are consistent with the authors’ observations and other articles describing these issues [102, 103]. It seems that effective lipid-lowering treatment, not only with statins but also with other drugs, improves the prognosis of COVID-19 patients. A study by Israel et al. assessed the effect of various medications taken by patients with COVID-19 on the course of the disease. It was shown that the use of rosuvastatin and ezetimibe significantly reduced the risk of hospitalization due to COVID-19 (OR = 0.673, 95% CI: 0.596–0.758, *p* < 0.001 and OR = 0.488, 95% CI: 0.377–0.622), *p* < 0.001, respectively) [104]. Barkas et al. suggest that proprotein convertase subtilisin/kexin type 9 (PCSK9) inhibitors, through their immunomodulatory properties, may reduce the risk of serious complications of COVID-19, such as acute respiratory distress syndrome and cytokine release syndrome [105]. It is indicated that PCSK9 reduces the expression of interferon β (INF-β) [106]. Therefore, the use of PCSK9 inhibitors, especially in patients with hypercholesterolemia, should reduce this adverse effect and improve the antiviral efficiency of the organism related to INF-β [107]. Overall, it has been suggested that lipid-lowering treatments, other than statins therapy, may also possibly reduce the severity of COVID-19, but this issue requires further research.

We strongly believe that optimal lipid-lowering therapy may improve the prognosis of patients with COVID-19. So, following the other recommendations we agree that strict control of lipid parameters in blood is of crucial importance especially during COVID-19 pandemic.

Compared to the previous ones, our article comprehensively presents the two-way relationship between lipid disorders and COVID-19. We critically discussed the cited research results and indicated the need to exercise caution when interpreting them. We have presented the latest meta-analyzes and clinical recommendations for the use of statins during the COVID-19 pandemic.

## Conclusions

Lipid disorders may increase the risk of a severe course of COVID-19, but on the other hand SARS-CoV-2 infection may itself cause lipid disorders in some patients, mainly impairing the function of HDL lipoproteins (which is obviously less important from the lclinical point of view). The use of statins may reduce the risk of severe caurse of COVID-19 and reduce the risk of death in these patients [108]. Statins, through their pleiotropic mechanism of action, can reduce the penetration of SARS-CoV-2 into the host cell and reduce the risk of complications from cytokine storm [109]. Patients with familial hypercholesterolaemia, and those being at high and very high CVD risk, may be at risk of the severe course of COVID-19. Therefore, lipid-lowering treatment should be particularly monitored in this group of patients.

A number of RCTs is currently underway to investigate the effect of statin use on COVID-19 score. More and more pathophysiological mechanisms linking lipidological problems with COVID-19 are under investigation, which will be finally answer all the existing questions on mechanisms and outcomes.

## Data Availability

Not applicable.

## Abbreviations

SARS-CoV-2: Severe acute respiratory syndrome coronavirus 2
COVID-19: Coronavirus disease 2019
RR: Relative risk
CVD: Cardiovascular disease
OR: Odds ratio
ACE2: Angiotensin converting enzyme 2
SR-B1: Scavenger receptor, class B type 1
LDL: Low density lipoprotein
HDL: High density lipoprotein
AIP: Plasma atherogenic index
BMI: Body mass index
PR: Prevalence ratio
IL-6: Interleukin 6
MCP-1: Monocyte chemoattractant protein-1
MIP: macrophage inflammatory proteins
PGE2: Prostaglandin E2
TXB2: Thromboxane B2
LTB4: Leukotriene B4
LXA4: Lipoxin A4
ApoA1: Apolipoprotein A1
ApoE: Apolipoprtoein E
SAA: Serum amyloid A
oxLDL: Oxidized low density lipoprotein
oxHDL: Oxidized high density lipoprotein
LOX1: Oxidized LDL scavenge receptor
sLOX1: Soluble oxidized LDL scavenge receptor 1
IL-10: Interleukin 10
TNFα: Tumor necrosis factor alpha
PON1: Paraoxonase 1
ABCA1: ATP-binding cassette transporter 1
LCAT: Lecithin–cholesterol acyltransferase
CETP: Cholesteryl ester transfer protein
LDL-R: Low density lipoprotein receptor
ApoCIII: Apolipoprotein CIII
LPL: Lipoprotein lipase
VLDL: Very-low density lipoprotein
CRP: C-reactive protein
HR: Hazard ratio
RCTs: Randomized controlled trials
SARS-CoV-2: Severe acute respiratory syndrome coronavirus 1
RNA: Ribonucleic acid
GM1: Monosialotetrahexosylganglioside 1
mRNA: Messenger ribonucleic acid
Mpro: Protease SARS-CoV-2
RdRp: RNA-dependent RNA polymerase
MAS: Macrophage activation syndrome
TLR-4: Toll-like receptor 4
NFκB: Nuclear factor kappa B
NLRP3: NOD-like receptor family, pyrin domain containing 3 inflammosome
EPCs: Endothelial progenitor cells
PAI-I: Plasminogen activator inhibitor-1
α-SMA: Alpha-smooth muscle actin
LOXL2: Lysyl oxidase-like protein 2
TGF-β: Transforming growth factor beta
EMT: Epithelial-mesenchymal transition
ESC: EUROPEAN Society of Cardiology
PCSK9: Proprotein convertase subtilisin/kexin type 9
INF-β: Interferon beta
